# Taking Impressions for Partial Sets

**Published:** 1869-03

**Authors:** W. N. Chaffee

**Affiliations:** Carlinville, Ill.


### ARTICLE VII.

### *Taking Impressions for Partial Sets.*

By W. N. Chaffee, Carlinville, Ill.

Every practitioner who is in the habit of taking plaster impressions will at once recognize the difficulty in the way of taking a perfect impression in plaster alone for a partial set, when the remaining natural teeth are long, and have large crowns, small necks and incline towards each other. Having adopted a method which overcomes the greatest obstacle, namely, that of removing the impression from the mouth entire, and without injury, I offer it for the benefit of others, not having seen or heard of its being used by any one.

We will take for example a case of the superior central incisors and bicuspids. I prepare my plaster as in other cases, having near at hand *warm* water for mixing it. Select an impression cup of suitable dimensions, preferring one with a square turned rim, instead of an oval one, as it accomodates the teeth much better. Prepare the cup by bridging across the back edge with a thin strip of wax not thicker than a knife blade to prevent the plaster from dropping over into the throat. I then soften some wax in warm water, making it as soft as possible without melting, and roll it out into a stick as large as my little finger, and long enough to pass around on the inside of the border of the cup from heel to heel. Heat the cup over a spirit lamp sufficiently to attach the wax to it, and lay the wax in around the border. The principal point to be observed in this method is expedition. After placing the wax in the cup, the plaster, mixed with *warm* water in order not to chill the wax, should be placed in the cup and introduced into the mouth as quickly as possible while the wax is in a plastic condition. Having my patient seated in an ordinary chair, and standing at his side, I pass my left arm over the head and hold it firmly in an upright position to prevent any plaster from dropping into the throat should any happen to fall over the back of the cup, with the right hand introduce the cup, press it well up, and hold it firmly until the plaster hardens. The wax takes the impression of the teeth very kindly and the plaster gives a perfect impression of the whole palatine surface.

After the plaster has become sufficiently hard the impression can be removed from the mouth without difficulty.

If the impression is for the four or six front teeth, then I do not let the wax extend all the way round the cup, but place it on each side, letting it extend just far enough to receive the impression of the teeth, leaving the front portion of the cup to be filled with plaster.

This method suggested itself in the following case:

About one year ago a patient applied to me for a partial set of teeth; on examining the mouth I found the remaining teeth long, much smaller at the gums than at cutting edge, and inclining towards each other. To obtain a perfect plaster impression was the object I had in view, for I considered a wax impression worthless in any case where an atmospheric pressure plate is required, I tried all the plans of which I had any knowledge, such as oiling the teeth, wrapping with cotton, placing wax in the spaces between them, &c., &c., but failed utterly to obtain anything like a perfect impression. After having made three or four unsuccessful attempts I discharged my patient, requesting her to call again the next day. In studying the case over in my mind that night I hit upon this experiment, I tried it and succeeded in obtaining a perfect impression the first time. Since then I have made use of it in all similar cases.

It occurs to me that there is a great need improvement in our impression cups.* I seldom attempt to take an impression for a full set, either upper or lower, without first fashioning my cup with wax, which I do in this way : I form a thin rim of wax all round the cup, I making it about one-eighth of an inch deeper. Before placing the plaster in the cup, warm it sufficiently to soften the wax, so that in pressing it into the mouth should the wax rim come in contact with the muscles of the cheek it yields readily, letting the cup to its place, which gives me a deep perfect impression embracing the whole alveoli.—*Missouri Dental Journal.*

* We are often reminded of this needed improvement in the form of impression cups. Will not some of our manufacturers give us something better ?

